# Clinical evaluation of a novel molecular diagnosis kit for detecting *Helicobacter pylori* and clarithromycin‐resistant using intragastric fluid

**DOI:** 10.1111/hel.12933

**Published:** 2022-10-20

**Authors:** Momoko Tsuda, Yoshiyuki Watanabe, Ritsuko Oikawa, Ryosuke Watanabe, Masayuki Higashino, Kimitoshi Kubo, Hiroyuki Yamamoto, Fumio Itoh, Mototsugu Kato

**Affiliations:** ^1^ Department of Gastroenterology National Hospital Organization Hakodate National Hospital Hakodate Japan; ^2^ Department of Internal Medicine Kawasaki Rinko General Hospital Kawasaki Japan; ^3^ Division of Gastroenterology, Department of Internal Medicine St. Marianna University School of Medicine Kawasaki Japan; ^4^ Department of Bioinformatics St. Marianna University Graduate School of Medicine Kawasaki Japan

**Keywords:** gastric juice, *H. pylori* diagnosis, PCR, point‐of‐care testing, susceptibility test

## Abstract

**Background:**

Although there are many *Helicobacter pylori* (*H. pylori*) diagnostic methods, the culture and antibiotic susceptibility test is an important method for selecting the most effective *H. pylori* eradication regimen. However, this diagnostic method is complicated and takes several days; therefore, the development of a rapid and simple diagnostic method is required. Eradication failure due to clarithromycin (CAM) resistance should also be considered. In this study, we report the clinical evaluation of point‐of‐care testing (POCT) kit using intragastric fluid, a novel kit for detecting *H. pylori and CAM resistance.*

**Materials and Methods:**

The study participants were 143 patients suspected of *H. pylori* infection and had an endoscopic examination. The novel diagnostic kit diagnosed *H. pylori* infection and CAM resistance‐associated mutation using intragastric fluid. To diagnose *H. pylori infection*, the relationship between the diagnostic kit and conventional diagnostic methods (urea breath test, stool antigen test, culture test, and real‐time polymerase chain reaction [PCR]) was evaluated. For CAM resistance‐associated mutation detection, the concordance between the diagnostic kit and antibiotic susceptibility test was evaluated.

**Results:**

The diagnosis of *H. pylori* infection with the novel molecular diagnostic kit using intragastric fluid showed significant relationship with conventional diagnostic methods. Especially when the culture was control, the sensitivity was 100% (67/67), the specificity was 95.9% (71/74), and the overall concordance was 97.9% (138/141). The detection of CAM resistance‐associated mutations had a concordance rate of 97.0% (65/67) when compared with the antibiotic susceptibility test.

**Conclusions:**

The *H. pylori* molecular POCT kit uses intragastric fluid as a sample and can diagnose *H. pylori* infection and detect CAM resistance‐associated mutations within an hour. This novel kit is expected to prove useful in selecting the most effective eradication regimen for *H. pylori*.

## 1 INTRODUCTION

*Helicobacter pylori* (*H. pylori*) infection causes various upper gastrointestinal disorders such as atrophic gastritis, gastric ulcer, duodenal ulcer, gastric cancer, gastric mucosa associated lymphoid tissue (MALT) lymphoma, and gastric hyperplastic polyp associated with chronic inflammation of the gastric mucosa.^1^ *H. pylori* infection is associated with gastric cancer as an important cause. The International Agency for Research on Cancer, the cancer research agency of the World Health Organization, strongly recommends designing a global preventive method for gastric cancer, including *H. pylori* eradication therapy.^2^, ^3^

In Japan, “*H. pylori*‐infected gastritis” has been included in the list of diseases covered by insurances since 2013. Consequently, eradication therapy for chronic gastritis is compensated by the national health insurance scheme. Interestingly, there has been a decrease in gastric cancer deaths since the start of the insurance coverage.^4^

The Japanese Society for *Helicobacter* Research guidelines recommend *H. pylori* eradication therapy as the primary preventive method for gastric cancer after antibiotic susceptibility test. Furthermore, the guidelines recommend the use of the therapy in two or three drug combinations to maximize the bacteria eradication rate because of possible eradication therapy failure.^1^ The failure of *H. pylori* eradication therapy is largely due to the resistance to clarithromycin (CAM), which is the primary drug for primary eradication. CAM resistance is caused by a gene mutation at positions 2142 and 2143 of the 23S rRNA gene Domain V region of *H. pylori*.^5^ We previously reported the applications of gastric wash sample (intragastric fluid), such as the detection of CAM resistance‐associated gene mutation of *H. pylori*, for selecting eradication therapy and quantitative analysis of *H. pylori* genotype via pyrosequencing analysis.^6^ This detection method using gastric wash sample (intragastric fluid) results in non‐invasive and high safety compared with the collect gastric mucosal biopsy for culture and susceptibility testing.^6^, ^7^ In addition, the results of antibiotic susceptibility testing take about 1 week; therefore, the method is too time‐consuming and impractical in clinical practice. Therefore, physicians need a new, time‐saving test to determine antibiotic susceptibility.

Smart Gene™ (MIZUHO MEDY Co., Ltd.) was developed on the concept of point‐of‐care testing (POCT), which can automatically perform nucleic acid extraction, amplification, and detection. The detection of *Mycoplasma pneumoniae* and coronavirus by Smart Gene™ with pharyngeal swabs shows high reproducibility and useability and allows quick patient triage.^8^, ^9^, ^10^, ^11^ In addition, the detection of *H. pylori* by Smart Gene™ with stool proved to be an effective non‐invasive test.^12^ The “*H. pylori* gene detection POCT reagent,” a reagent for the fully automatic Smart Gene™, allows for the automatic detection of *H. pylori* and CAM resistance‐associated mutations based on the Qprobe method.^13^, ^14^, ^15^

Our goal was the fast and accurate diagnosis of *H. pylori* and detection of CAM resistance for the purpose of determining a precise eradication therapy. Therefore, we evaluated the clinical performance of the “*H. pylori* molecular POCT kit” in diagnosing *H. pylori* infection and detecting CAM‐resistant mutations. In this paper, we report new fast and accurate clinical technique for the diagnosis *H. pylori* infection in as fast as about an hour with the kit by Smart Gene™.

## 2 MATERIAL AND METHODS

### 2.1 Patient enrollment and *Helicobacter pylori* conventional testing

**FIGURE 1:**
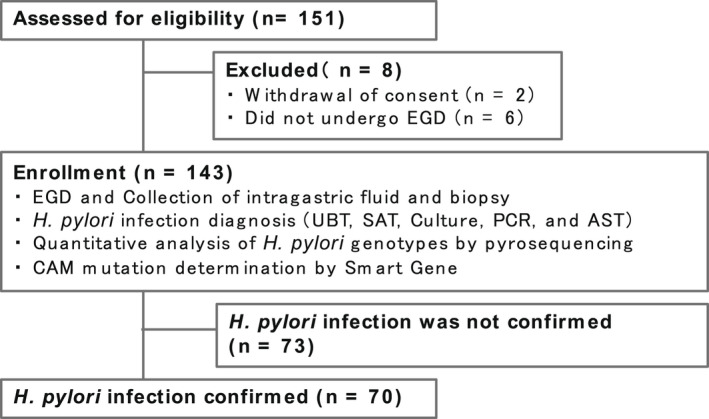
The flowchart of participants. We finally enrolled 143 patients for whom we performed endoscopic examination to detect *Helicobacter pylori* infection and clarithromycin‐resistant mutation by conventional testing, pyrosequencing, and *H. pylori* gene measurement. AST, Antibiotic susceptibility test; EGD, esophagogastroduodenoscopy; PCR, Polymerase chain reaction; SAT, Stool antigen test; UBT, Urea breath test

To collect samples through esophagogastroduodenoscopy (EGD) for this study, we assessed the eligibility of 151 patients suspected of *H. pylori* infection at the National Hospital Organization Hakodate Hospital and the Kawasaki Rinko Hospital from December 2019 to March 2021. However, a total 8 patients were excluded from the study because they withdraw their consent (*n* = 2) or did not undergo EGD (*n* = 6); therefore, 143 patients were enrolled in the study (Figure 1). In addition to EGD, selected patients underwent a urea breath test (UBT, Urea breath test, UBIT tablet, 100 mg; Otsuka Pharmaceutical Co., Ltd.) stool antigen test (SAT, Stool antigen test, Testmate rapid pylori antigen; Wakamoto Pharmaceutical Co., Ltd) and *H. pylori* culture test (BML Co., Ltd.) as part of the conventional tests required for the diagnosis of *H. pylori* infection. From the result of the tests, 70 patients were confirmed to be *H. pylori‐*positive and 73 patients were confirmed to be *H. pylori*‐negative.

### 2.2 Sample collection of gastric mucosal biopsy and intragastric fluid

**FIGURE 2:**
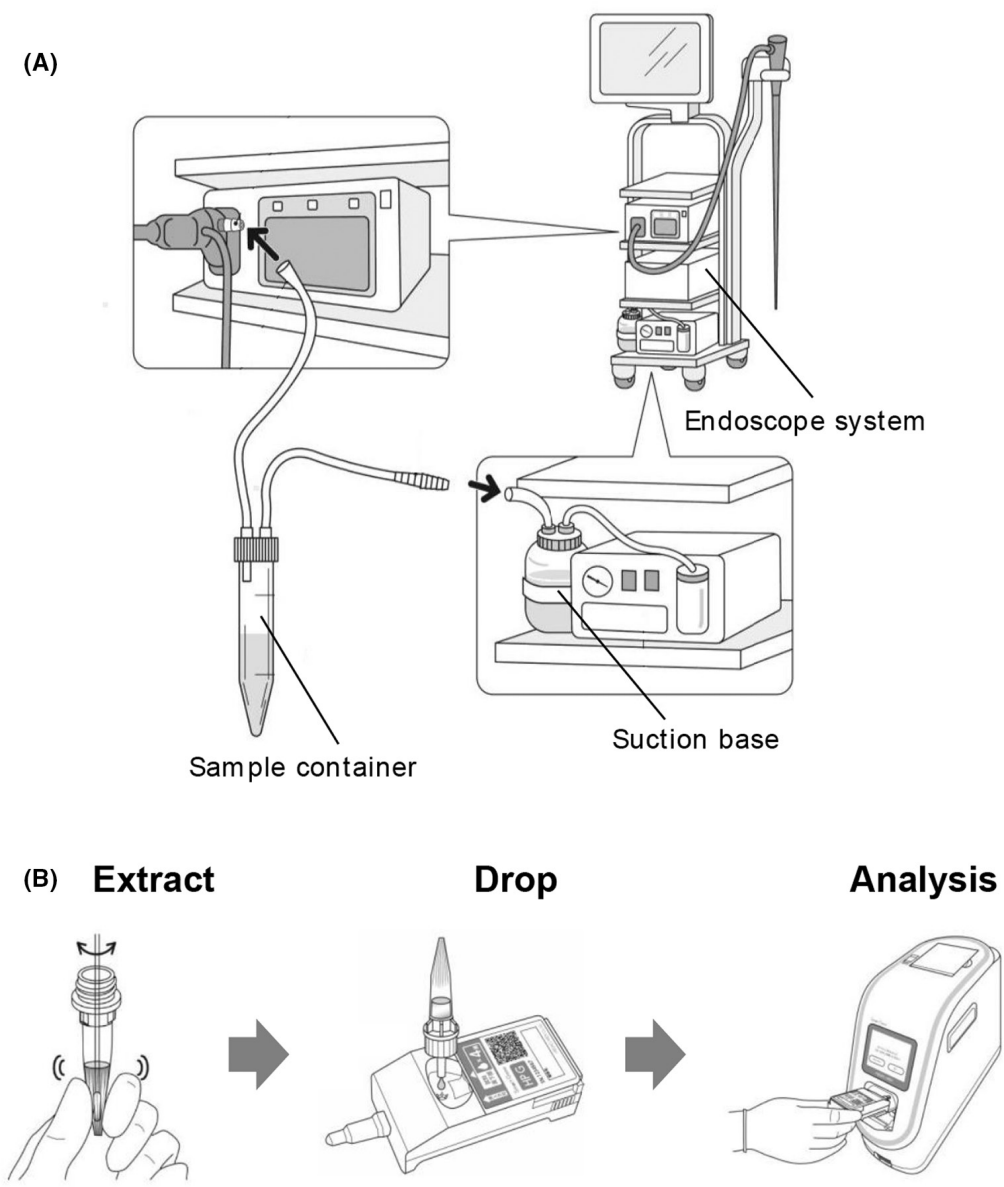
(A) Collection of intragastric fluid using sample container. (B) Assay workflow for *Helicobacter pylori* molecular POCT kit with Smart Gene™

To diagnose *H. pylori* infection and antibiotic susceptibility for CAM, we collected the patients' intragastric fluid and performed gastric mucosal biopsy as part of the endoscopic examination procedure, which has been previously reported.^6^ Patient drank 100 ml of water (including 80 mg dimethylpolysiloxane, 1 g sodium bicarbonate, and 20,000 units of pronase) 10 min prior to their endoscopy. We collected a sample tissue of approximately 5 mm under endoscopic observation using biopsy forceps. Patients' intragastric fluid was collected directly to a sample container (MD‐33050, SB‐Kawasumi Laboratories, Inc.) through the connected suction base of the endoscope (Figure 2A).

### 2.3 *Helicobacter pylori* antibiotic susceptibility test

To determine *H. pylori* susceptibility, we performed antibiotic susceptibility testing of CAM using gastric mucosal biopsy as a conventional diagnosing method. *The* susceptibility tests were conducted by the clinical testing contractor BML Co., Ltd. In detail, gastric biopsies were cultured on Nissui Plate Helicobacter agar (Nissui Pharmaceutical Co., Ltd.) under microaerophilic conditions (5% O_2_, 15% CO_2_, and 80% N_2_) for 4 days at 37°C. Then, the isolated 3 colonies were enriched on 5% sheep blood agar (Nippon Becton Dickinson Co., Ltd.). The enriched colonies were prepared to a McF1.0 bacterial solution, and 25 μl bacterial solution was added to 6 ml of 10% horse serum‐added Mueller Hinton broth (Nikken Biological Co., Ltd.) to prepare a sample. Antibiotic susceptibility testing of CAM was performed using the dry plate “Eiken” (Eiken Chemical Co., Ltd.). The antibiotic susceptibility of CAM was determined using 1 μg/ml, minimal inhibitory concentration (MIC) breakpoint, recommended by the Japan Society of Chemotherapy and the Clinical & Laboratory Standards Institute (CLSI).^16^, ^17^

### 2.4 Smart Gene assay for detection of *Helicobacter pylori* DNA and CAM‐resistant mutation

To evaluate the effectiveness of the novel “*H. pylori* molecular POCT kit” in detecting *H. pylori* and CAM‐resistant mutations using Smart Gene™ (Mizuho Medy Co., Ltd.), we used intragastric fluid as a sample. We simply dropped intragastric fluid suspended into the test cartridge “*H. pylori* molecular POCT kit.” Smart Gene™ detects the *H. pylori* DNA and CAM‐resistant mutations at positions 2142 and 2143 of the 23S rRNA gene based on polymerase chain reaction (PCR) and QProbe. The principle of detecting gene mutations by QProbe is based on previous report that demonstrated use of the macrolide‐resistant *Mycoplasma pneumoniae* (MRMp) diagnostic kit.^10^ The use of Smart Gene™ allows for the simultaneous diagnosis of *H. pylori* infection and detection CAM resistance‐associated mutation. In our study, the Smart Gene™ automatically processed the samples and generated the results within an hour. In addition, because we utilized intragastric fluid, there was no additional invasive procedure carried out on study participants at the time of endoscopy (Figure 2B).

### 2.5 Real‐time PCR of *Helicobacter pylori* 16S rRNA

To compare the outcomes of different testing methods, we performed a molecular diagnostic real‐time PCR targeting the *H. pylori* 16S rRNA gene as our control.^18^ We extracted DNA from 200 μl intragastric fluid with the QIAamp DNA Mini Kit (QIAGEN GmbH) to obtain 150 μl purified DNA. The forward primer CGC‐TAA‐GAG‐ATC‐AGC‐CTA‐TGT‐CC and the reverse primer CCG‐TGT‐CTC‐AGT‐TCC‐AGT‐GTG‐T were used for real‐time PCR. Real‐time PCR was performed using the Thermal Cycler Dice Real Time System III instrument (Takara Bio) using TB Green Premix Dimer Eraser Perfect Real Time reagent (Takara Bio) under the following PCR conditions: preheating at 95°C for 30 s, and 50 cycles at 95°C for 5 s and at 55°C for 30 s and at 72°C for 30 s. The PCR amplicon was confirmed by melting curve analysis.

### 2.6 Quantitative analysis of *Helicobacter pylori* CAM‐resistant mutation ratio by pyrosequencing

To assess and determine CAM resistance, we quantitatively analyzed mutation rates at positions 2142 and 2143 of the *H. pylori* 23S rRNA gene via pyrosequencing, adopting previously reported procedures.^6^ One milliliters intragastric fluid was centrifuged at 1200 *g* for 15 min and 800 μl supernatant was decanted. 200 μl purified DNA was extracted from residue sample with the QIAamp DNA Mini Kit (QIAGEN GmbH). Thereafter, the *H. pylori* 23S rRNA gene was amplified and biotinylated by nested PCR. In the first reaction, a 255‐bp fragment was amplified with the forward primer ACG‐AGA‐TGG‐GAG‐CTG‐TCT‐CAA‐CC and the reverse primer AGC‐ATT‐GTC‐CTG‐CCT‐GTG‐GAT‐AAC. The amplified fragments were, thereafter, used as a template in the second reaction to amplify a 90‐bp fragment with the forward primer GAG‐GTG‐AAA‐ATT‐CCT‐CCT‐ACC‐CGC‐G and the reverse primer GCG‐CAT‐GAT‐ATT‐CCC‐ATT‐AGC‐AGT‐GC. Reactions consisted of touchdown PCR with denaturation at 95°C for 30 s, annealing at appropriate temperatures for 30 s, and extension at 72°C for 30 s. Finally, the amplified fragments were analyzed by pyrosequencing on a Pyromark Q24 system (QIAGEN) using primer ACC‐CGC‐GGC‐AAG‐ACG.

### 2.7 Statistical analysis

Concordance between Smart Gene assay and conventional diagnosis was evaluated by Cohen's kappa coefficient. All statistical analyses were conducted using the R 4.2.1 software program (www.r‐project.org).

## 3 RESULTS

### 3.1 Evaluation of “*Helicobacter pylori* molecular POCT kit” by Smart Gene™ in *Helicobacter pylori* infection diagnosis

Results from the use of the “*H. pylori* molecular POCT kit” by Smart Gene™ was strongly correlated with the results from conventional *H. pylori* diagnosing methods. To evaluate the diagnostic accuracy of “the molecular PCOT kit,” conventional *H. pylori* diagnostic methods, such as UBT, SAT, culture test, and PCR, were compared (Table 1). The culture test, with a sensitivity of 100% (67/67) and specificity of 95.9% (71/74), showed the highest concordance rate with POCT kit.

**TABLE 1:** Comparison of Smart Gene™ assay using intragastric fluid and conventional diagnosis of *Helicobacter pylori* infection

Control method	Detection of *H. pylori* DNA by Smart Gene™				
	Positive	Negative	Sensitivity (%)	Specificity (%)	Cohen's κ
UBT					
Positive	64	5	92.8	94.4	0.871
Negative	4	67			
SAT					
Positive	60	1	98.4	88.9	0.865
Negative	8	64			
Culture					
Positive	67	0	100	95.9	0.957
Negative	3	71			
PCR					
Positive	69	3	95.8	98.6	0.944
Negative	1	70			

Abbreviations: PCR, polymerase chain reaction; SAT, stool antigen test; UBT, urea breath test.

### 3.2 Discrepancy in the diagnosis of *Helicobacter pylori* by Smart Gene

The mismatch of “*H. pylori* molecular POCT kit” by Smart Gene™ from conventional *H. pylori* diagnosis methods occurred in 19 cases in this study. (Table 2). In most cases, the results of POCT kit did not match completely. However, in several test results, except Case 6, POCT kit gave different results. In Case 6, only the results of POCT kit showed positive. Three cases (Case 4, 5, 13) tested positive for only PCR but negative for other methods including POCT kit.

**TABLE 2:** List of the mismatch cases between Smart Gene™ assay and control methods

Case No.	Control method					Smart Gene
	UBT	‰	SAT	Culture	PCR	
1	+	10.3	N.D.	−	−	−
2	+	5.8	−	−	+	+
3	−	0.5	+	−	−	−
4	−	1.4	−	−	+	−
5	−	0.6	−	−	+	−
6	N.D.	N.D.	−	−	−	+
7	+	6.2	−	−	−	−
8	+	2.6	−	−	−	−
9	+	68.7	−	+	+	+
10	+	9.5	−	+	+	+
11	−	2.1	+	+	+	+
12	−	1.4	+	−	+	+
13	−	1.9	−	−	+	−
14	−	1.2	−	+	+	+
15	+	2.8	−	−	−	−
16	+	3.1	−	−	−	−
17	−	1.7	−	+	+	+
18	+	57.7	−	+	+	+
19	+	6.4	−	+	+	+

Abbreviations: N.D., no data; PCR, polymerase chain reaction; SAT, Stool antigen test; UBT, UREA BREATH test.

### 3.3 Comparison of CAM resistance and the mutation ratio of *Helicobacter pylori* 23S rRNA by pyrosequencing

**FIGURE 3:**
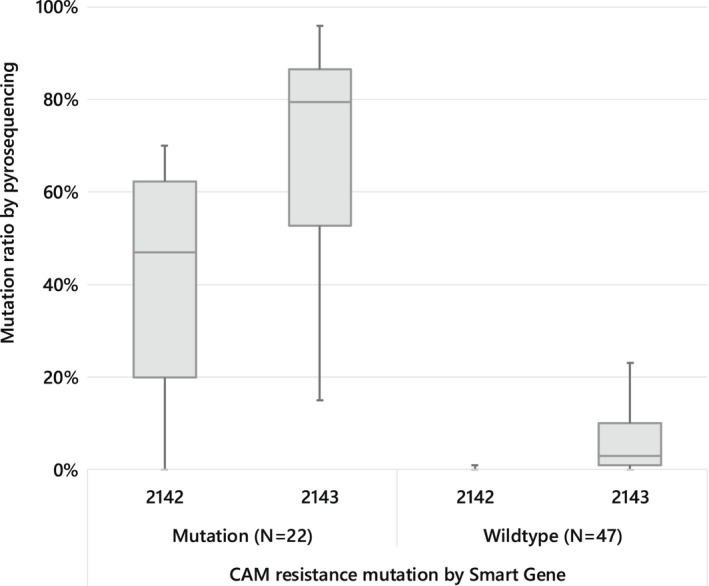
Comparison of CAM resistance determined by Smart Gene™ assay and mutation ratio of *Helicobacter pylori* 23S rRNA gene by pyrosequencing. CAM, clarithromycin

Quantitative pyrosequencing analysis of mutation rates at positions 2142 and 2143 of the *H. pylori* 23S rRNA gene supported the results of “*H. pylori* molecular POCT kit” by Smart Gene™ in detecting clarithromycin resistance up to a mutation rate of about 20%. The results of POCT kit were compared with quantitative pyrosequencing analysis of the mutation rates of *H. pylori* 23S rRNA genes at positions 2142 and 2143. Regardless of their locations, all the mutations at position 2142 detected by pyrosequencing analysis were also detected as mutations by POCT kit. On the contrary, although mutations were detected by pyrosequencing analysis at position 2143, POCT kit could only detect the mutations at position 2143 if they were 20% or more (Figure 3). For the mutation at position 2143, the minimum mutation rate determined as a mutation by POCT kit was 15%, while the maximum mutation rate determined as a wild type by POCT kit was 23%.

### 3.4 Evaluation of the detection of clarithromycin resistance using “*Helicobacter pylori* molecular POCT kit” by Smart Gene™

“*Helicobacter pylori* molecular POCT kit” by Smart Gene™ was able to detect clarithromycin resistance up to the mutation rate of about 20% by pyrosequencing. In determining clarithromycin resistance, the concordance rate for resistance was 91.7% (22/24) and the concordance rate for susceptibility was 100% (43/43) between Smart Gene™ and the control test, antibiotic susceptibility test (AST). The overall match rate was 97.0% (65/67; Table 3). Two discrepancy results represented 13% and 23% in the mutation rate through quantitative pyrosequencing analysis, which are low mutation rates.

**TABLE 3:** Comparison of CAM resistance determined by Smart Gene™ assay and AST

Control method	Detection of CAM resistance mutation by Smart Gene™				
	Mutation	Wild type	Sensitivity	Specificity	Cohen's κ
AST					
Resistant	22	2	91.7%	100%	0.934
Sensitive	0	43			

Abbreviation: AST, Antibiotic susceptibility test.

## 4 DISCUSSION

“*Helicobacter pylori* molecular POCT kit” by Smart Gene™ allows physicians to safely collect specimens and obtain diagnostic results on time. The simplicity of the kit allows for fast and accurate diagnosis of CAM resistance in determining a patient's precise eradication therapy. Consequently, its wider application will lead to more patients being tested for their *H. pylori* infection status.

The effectiveness of the “*H. pylori* molecular POCT kit” in detecting a CAM resistance‐associated mutation rate at a high concordance rate as an antibiotic susceptibility test proves beneficial in daily clinical practice. This novel kit can detect gene mutation at positions 2142 and 2143 of the 23S rRNA gene domain V region of *H. pylori*, precisely indicating CAM resistance. Furthermore, the “*H. pylori* molecular POCT kit” can detect CAM resistance‐associated mutations at a mutation rate of 20% or more when compared to pyrosequencing analysis. Results obtained by “*H. pylori* molecular POCT kit” in detecting CAM resistance is a great indication of patients' eradication results.

Using “*H. pylori* molecular POCT kit” for detecting CAM‐resistant mutation can significantly improve time‐efficiency in planning a specific treatment plan for patients. Due to its simplicity and speed, compared with other existing antibiotic susceptibility tests, physicians can speed up the process of selecting the appropriate antibiotic for the eradication of *H. pylori*, allowing patients to receive treatment as early as possible. As a result of the quick results provided by “*H. pylori* molecular POCT kit,” physicians can provide individualized treatment for patients with CAM resistance, such as skipping CAM‐based therapy and jumping into non‐CAM‐based therapy. Consequently, this will save physicians a significant amount of time they would have wasted on waiting for results and trying out ineffective treatments.

Additionally, “*H. pylori* molecular POCT kit” is safer for patients than conventional tests because it requires no additional invasive procedure during endoscopic examination and offers painless procedures. Patients only need to provide their intragastric fluid as a test specimen and do not have to go through gastric mucosal biopsy, which could cause complications, including excessive bleeding. Furthermore, because collecting intragastric fluid requires very little preparation time, patients do not have to suffer any pain, which they are likely to experience with longer preparation time. This significantly lower the risk of patient's vital transition.

The ease‐of‐use and reliability of results with the “*H. pylori* molecular POCT kit” could lead to the consolidation of all the different *H. pylori* diagnostic tests currently in use. Although the occurrence of false positives and negatives need to be further investigated, the kit has a high likelihood of replacing existing diagnostic tests. In this study, three cases (Case 4, 5, 13) tested positive for only PCR assumed to be the result of insufficient *H. pylori* amount.

“*H. pylori* molecular POCT kit” is so simple and so capable of accurately and safely diagnosing *H. pylori* infection that it could be a great alternative diagnostic method in near future. Because the kit has a wide application and allows for the simultaneous testing of *H. pylori* infection and CAM resistance statuses, physicians can save time on eradication failure caused by CAM resistance.

One limitation of our study is the sample size. Although our sample size was sufficient enough to give us acceptable data, to increase the accuracy of the kit, we would like to enroll and study more people diagnosed with *H. pylori* infection. Because participating the study and eradicating *H. pylori* only lower participants' risk of developing gastric cancer, this study is also a part of eradicating overall gastric cancer itself.

## AUTHOR CONTRIBUTIONS

MT performed the experiments and analyses and drafted the manuscript. YW performed the experiments. RO performed pyrosequencing. RW, MH, and KK performed the experiments. HY and FI supervised the experiments. MK supervised the entire project.

## FUNDING INFORMATION

MIZUHO MEDY Co., Ltd provided grants and *H. pylori* molecular POCT kit for this study, but played no role in the study design, data collection and interpretation, or in the decision to submit the work for publication.

## CONFLICT OF INTEREST

The authors declare no conflict of interests associated with this study.
